# The Impact of Physical and Ergonomic Hazards on Poultry Abattoir Processing Workers: A Review

**DOI:** 10.3390/ijerph13020197

**Published:** 2016-02-06

**Authors:** Johannes L. Harmse, Jacobus C. Engelbrecht, Johan L. Bekker

**Affiliations:** Department of Environmental Health, Tshwane University of Technology, Private Bag X680, Pretoria 0001, South Africa; Engelbrechtjc@tut.ac.za (J.C.E.); Bekkerjl@tut.ac.za (J.L.B.)

**Keywords:** poultry abattoir processing, ergonomic, work related upper limb disorders, noise, cold, poultry processing health effects, occupational exposure

## Abstract

The poultry abattoir industry continues to grow and contribute significantly to the gross domestic product in many countries. The industry expects working shifts of eight to eleven hours, during which workers are exposed to occupational hazards which include physical hazards ranging from noise, vibration, exposure to cold and ergonomic stress from manual, repetitive tasks that require force. A PubMed, Medline and Science Direct online database search, using specific keywords was conducted and the results confirmed that physical and ergonomic hazards impact on abattoir processing workers health, with harm not only to workers’ health but also as an economic burden due to the loss of their livelihoods and the need for treatment and compensation in the industry. This review endeavours to highlight the contribution poultry processing plays in the development of physical agents and ergonomic stress related occupational diseases in poultry abattoir processing workers. The impact includes noise-induced hearing loss, increased blood pressure, menstrual and work related upper limb disorders. These are summarised as a quick reference guide for poultry abattoir owners, abattoir workers, poultry associations, occupational hygienists and medical practitioners to assist in the safer management of occupational health in poultry abattoirs.

## 1. Introduction

Globally, the poultry sector continues to grow in terms of production, as well as number of employers, due to the increasing human population, an increased demand for animal protein, its healthy label, affordability, greater consumer purchasing power, product variation and urbanisation [1]. The major broiler producers manage integrated broiler meat supply chains which include the production of day-old chicks, broiler farms, feed milling, meat processing and distribution to customers [2,3]. The industry is a major contributor to the gross domestic product and to society at large. The Food and Agriculture Organisation of the United Nations (UN) estimates an annual growth of 1.6% in the industry globally to produce some 108.7 million tonnes of poultry meat [4]. In South Africa (SA), the poultry industry is the country’s largest individual agricultural industry contributing 17% to the gross value of agricultural products with an annual growth of 1.3% in 2013 [2]. The food sector employs around 22 million workers worldwide in food and drink manufacturing, a figure which may increase significantly if jobs throughout the entire food production system are counted [5,6].

Food production industries worldwide are experiencing a constant rise in standards to ensure food quality and food safety, for example, the ISO 22 000 of 2005: Food Safety Management System which is imposed by retail and benefits business and creates opportunities [7,8]. Conversely, the constant drive for higher profit and production, as well as increasing production line speeds, impact negatively on working conditions [9,10]. United States (U.S.) unions, such as the Food, Agricultural, Hotel, Catering and Allied Workers Union and the National Union of Workers, state that safe food begins with worker safety and health and that unlawful and unethical practices at production facilities are reducing and compromising the quality and safety of food produced [11,12,13].

The objective of this review is to present the extensive role that poultry abattoir processing plays in the development of physical and ergonomic related health impacts on workers’ health. In order to achieve the objective, the paper addresses the aspects of poultry meat production, occupational impacts and diseases, applicable legislation and the management of ergonomic and physical risk.

Disease agents, hosts and the work environment is an ecosystem that is in dynamic balance, but when occupational exposure occurs disease agents impact on the health of the host and disturb this balance causing occupational disease [14,15,16]. According to the International Labor Organization (ILO), the U.S. Accountability Office and the UN Human Rights Watch (HRW), workers in the poultry abattoir processing industry are exposed to several occupational health hazards namely:

- physical agents such as noise, exposure to cold, vibration [17,18,19];
- ergonomic hazards including manual and repetitive work such as hanging and cutting, forceful exertion, awkward work positions and fast work pace [17,18,19];
- hazardous chemical substances including dust, cleaning/disinfecting chemicals, value adding products and gases [17,18,19];
- hazardous biological agents such as bacteria, viruses, fungi, endotoxins and ectoparasites [17,18,19].

The HRW reports that poultry processing workers perform one of the most dangerous jobs and the work environment poses risks greater than those faced by workers in many other manufacturing processes and sectors [20]. In addition to impacting on worker health, exposure may impact on absenteeism, reduce the quality of life of employees and compromise productivity and product quality [21]. According to the HRW, the poultry industry sets up facilities and introduces practices which create hazards and risks to workers and treat the resulting mayhem as a normal natural part of the production process and not as possible violations of international human rights and many national constitutions. Work practices are often in conflict with UN principles which state that everyone is entitled to the enjoyment of favourable, safe and health conditions at work [20,22]. Conditions which typically develop include blood pressure and menstrual disorders, noise-induced hearing loss, hypothermia, frostbite and ergonomic effects including work-related upper limb disorders (WRULD), which is a collective term used to describe diseases of the musculature and skeleton such as rotator cuff syndrome, epicondylitis at the elbow, tenosynovitis and nerve entrapments such as carpal tunnel syndrome.

### 1.1. Legal Control

The ILO support national frameworks and policies in occupational health and safety management and ILO member countries are required to support their mission, vision, goals and objectives by implementing occupational health management systems on a national level. SA was a member from 1919 until 1966 and then from 1994 to date [23,24,25,26]. International as well as national occupational health legislation places the burden of worker health on the employer and in support of this, legislation such as the Occupational Health and Safety Act of 1995 in SA and the Health and Safety at Work Act of 1974 in the United Kingdom (UK) requires employers to provide a healthy workplace [27,28]. From literature sourced, no poultry specific occupational health legislation exists and generic occupational health legislation applies. Literature confirms that some of the occupational health aspects, for instance vibration and manual handling, is not legislated in SA, thereby leaving workers at a disadvantage [29,30], as reflected in Table 1.

**Table 1:** Legislation available for physical agents and ergonomic occupational hazards and compensation status.

Physical Agent	Legislation/Regulation			Compensable Disease		
	SA	U.S.	UK	SA	ILO	UK
Noise	Noise-induced hearing loss regulations, 2003	Occupational noise exposure regulations 1910:95	The control of noise at work regulations, 2005			
	**NOISE OEL**					
	L_Ar_8 _hr_**^1^** < 85 dB(A) **^2^**	Equivalent noise level should be < 90 dB(A) for 8 h also sets: —Action level: 8 h TWA **^3^** of 85 dB(A) or 50% noise dose	Daily or weekly personal noise exposure of 87 dB(A) & a peak Lp **^4^** not > 140 dB(C) also sets: —Lower exposure action value: Daily or weekly exposure of 80 dB(A) & peak Lp 135 dB(A); —Upper exposure action values: Daily or weekly exposure of 85 dB(A) & peak Lp of 137 dB(A)			
Cold	Environmental regulations for workplaces, 1987	Occupational safety and health act, 1970 —Occupational safety and health standards 1910:999	Workplace regulations, 1992			
	**COLD OEL**					
	The four hour TWA Dry-bulb temperature index should not exceed 6 °C	The Wind-Chill Index is used prescribing maximum exposure times at certain wind chill temperatures	Dry-bulb temperature and air velocity used to determine the Wind Chill Factor —Several OELs provided			
Vibration	Nil	Occupational noise exposure regulations 1910:95	Control of vibration at work regulations, 2005			
	**VIBRATION OEL**					
	Nil	ACGIH set an acceleration of 4 m/s^2^ for 4–8 h, dropping to 8 m/s^2^ for 1–2 h	Acceleration as Action limit of 2.5 m/s^2^ and an OEL of 5.0 m/s^2^			
Ergonomic hazards	OHSACT **^5^**, 1993: General duty clause	OSHACT **^6^**, 1970: General duty clause	Manual handling operations regulations, 1992			
	**ERGONOMIC OEL**					
	Nil General duty clause	ACGIH **^7^**: —hand activity tab les for hands & wrists based on repetitive-ness & force used —screening & lifting for lower back problems	MAC **^8^** tool, ART **^9^** tool			

**^1^** L_Ar_8 _hr_—8 h noise rating level; **^2^** dB(A)—Decibel in the A scale; **^3^** TWA—Time weighted average; **^4^** Lp—Sound pressure level; **^5^** SA—Occupational Health and Safety Act; **^6^** U.S.—Occupational Safety and Health Act; **^7^** American Conference for Governmental Industrial Hygienists; **^8^** MAC—Manual handling assessment charts tool; **^9^** ART—Assessments of repetitive tasks tool.

Occupational Exposure Limits (OEL) are set to ensure exposure does not affect worker health and are based on the principle that exposure should be as low as is reasonably possible and should assist in preventing occupational disease [31]. 

Table 1 provides a summary of physical agents and ergonomic occupational hazards in poultry abattoirs, national and international legislation of some countries, if effects are compensable, as well as the applicable OEL [16,17,27,28,32,33,34,35,36,37,38,39,40,41,42].

### 1.2. Reporting of Occupational Disease

All ILO member countries must have systems in place to report and compensate workers. The reporting and compensation of occupational diseases in SA is addressed by the Compensation for Occupational Injuries and Diseases Act of 1993 and in the UK, by the Reporting of Injuries, Diseases and Dangerous Occurrences Regulations of 2013 [28,36]. 

**Table 2:** Effects of physical agents on workers.

Physical Agents in Poultry Abattoirs		
Effect	Notes	References
**Noise**		
1994: 1997: Temporary or permanent hearing loss	Contributing factors: Age, obesity, workplace size, irregular shifts, production line work	[43,44]
2008: Noise-induced hearing loss (NIHL)	Noise levels > 80 dB(A) presented NIHL levels > 20%. Bilateral hearing damage at 3, 5 and 6 Kilohertz cause NIHL ranging 15–50 dB **^1^**	[45]
1997: Negative impact on communication	Misinterpretation of messages	[43]
1983: 1985; 1990: Chronic arterial hypertension	Blood pressure increases exponentially with every 5 dB (A) increase in women	[46,47,48]
1995: Reproduction risks	Affect foetus, low birth weight, reduced gestation period, foetal loss	[49,50]
1995: Menstrual disturbances	In female poultry processors	[49]
2008: Lower productivity	Increase in absenteeism due to illnesses at 80 dB(A)	[45]
2008: >12% increase in accidents due to higher noise levels		[45]
1984: Accident frequency increased in noise areas	Lower levels beneficial to productivity, product quality	[51]
**Vibration**		
1997: Raynaud’s syndrome in poultry abattoir processing workers	Increase finger sensitivity; Syndrome More prevalent in women; Link with cold and repetition	[52]
**Cold**		
2012: Cooling of hands	Significant productivity drop Pain, numbness, skin damage	[53]
2004: Back and neck pain	At 2 °C	[54]
2011: Hypothermia **^2^** and death	Speech impediment, shiver, confusion Aggravates MSD	[55,56]
2012: Increase in accidents	Hypothermia	[56]
1996: Frost bite	Skin burns and damage	[17]
1985: Dysmenorrhea **^3^**, Irregular menstrual cycles	Link between cold and Dysmenorrhea with respect to age, parity, oral contraceptive use	[57]
1992: Amenorrhea **^4^**	Prevalent in 12% female poultry workers Absenteeism increase	[58]

**^1^** dB—Decibel: Linear unit for noise measurement; **^2^** Body temperature dropping below 35.7 °C; **^3^** Painful menstruation; **^4^** Abnormal absence of menstruation.

The legislation in Table 2 provides for the controlling of occupational exposure to prevent disease and for the reporting of occupational diseases, including within SA. WRULDs is a collective term for a group of occupational diseases that consist of musculoskeletal disorders (MSD), caused by exposure in the workplace, affecting the muscles, tendons, nerves, blood vessels, joints and bursae of the hand, wrist, arm and shoulder caused by repetitive movement. These syndromes are associated with symptoms and physical signs including pain, swelling and difficulty in moving. It includes nerve entrapments such as carpal tunnel syndrome (CTS), tenosynovitis, epicondylitis (elbow), tendonitis, bursitis and trigger finger [59]. Outdated terminology, such as repetitive strain injury (RSI) and cumulative trauma disorder (CTD), are no longer recognised as the term MSD is accepted as encompassing all conditions of the musculature and skeleton, and the collective term WRULDs is preferred [60,61,62].

The basis for compensating workers for WRULD is complex and varies greatly between countries, with conditions not solely attributable to work such as trigger finger, Raynaud’s syndrome, myalgia neuropathies and others not included as compensable as solely attributable to work [59,63,64].

MSD, including CTS, represents the most common work related health disorder in 27 European Union (EU) countries representing 59% of all recognised diseases during 2005 [25]. During 2011/2012, MSD represented 40% of all work related cases across all sectors [25]. With 80% prevalence, MSD, together with work stress and anxiety, tops the list for work related ill health across all sectors. In the UK, MSD accounts for 526,000 out of 1,241,000 cases. The number of new cases of MSD in 2013/2014 was 184,000, up from 141,000 in 2011/2012 [65]; the total number of working days lost due to MSDs in 2013/2014 was 8.3 million, an average of 15.9 days per case of MSDs across all sectors [40]. Meat processors are among the more exposed occupations for upper limb disorders from repetitive work [66]. Concerning the food production sector, the Health and Safety Executive (HSE), in 2004, identified musculoskeletal disorders (MSDs), mainly comprising work-related upper limb disorders (WRULDs) and back injuries, and noise-induced hearing loss (NIHL) as the top UK occupational diseases [67]. The HSE classifies poultry production as a sector of concern reflecting increasing occupational disease and injury rates [68,69]. 

In the U.S., civic organisations and worker unions claim the injury rate is almost twice as high for workers in poultry processing, at 5.9%, compared to that of workers in the private sector which were at 3.8%. This statement was refuted by United States Poultry and The National Chicken Council [70,71].

Compensable cases for poultry workers between 1985 and 1992 increased from 1196 to 1928. Incidence rates per 100 employees per year were highest during 1978, at 3.25, followed by 3.11 in 1990. Forty one percent of the workers compensated have worked less than one year. Strains and sprains accounted for the highest percentage of cases (41%) with the back being the most frequently affected body part (66%) and more than one third (36%) of all cases occurring to the upper extremities. A relationship between workers’ compensation costs and lost workdays has been determined [72].

The ILO is however of the opinion that under-reporting of compensable occupational diseases often occur, an opinion which is shared by the HSE [23,25,73,74,75,76,77,78]. Occupational disease may go unrecognised because:

- diagnosing occupational injuries such as broken limbs or cuts is less complicated than diagnosing asthma, allergies or inflammation which develops slowly or away from the workplace and might have multiple causes and linking disease to causation might require specialised skill [79,80];
- bonuses are often linked to injury and production rates making it contradictive to a healthier workplace [81];
- company operated clinics are seen by workers as an extension of management and workers claim clinics fail to take injuries seriously by often stating that workers are looking for excuses not to work [73,82].

Underreporting of non-fatal occupational health and safety accidents and diseases across all U.S. industry sectors is estimated at 69% [83]. Companies only report work days lost and workers are often re-assigned to other tasks and the incidence or disease is never reported. Worker interviews by HRW show substantial underreporting of musculoskeletal disorders in ill or injured workers in the U.S. poultry industry; no such statistics exist for SA. To highlight this phenomenon, some worker comments on reporting are reflected below. HRW recorded that all workers interviewed for this report bore physical signs of a serious injury suffered from working. Their accounts of life in the factories graphically explained those injuries. Automated lines move too fast for worker safety. Repeating thousands of cutting motions during each work shift puts enormous traumatic stress on workers’ hands, wrists, arms, shoulders and backs. They receive little training and are often forced to work long overtime hours under threat of dismissal if they refuse [20]:

- “The company hates to report any incidents (incident—an accident or a near-miss event where no injury or illness occurs) that occur at poultry abattoirs to the U.S. Occupational Safety and Health Authority (OHSA)”;
- “You work like a dog and when you get hurt you are trash”;
- “If you get hurt they will look for a way to get rid of you before they report it, they find a reason to fire you or put you in the worse job like the cold room, or they change your shift so that you quit. It is better just work with the pain and don’t report it”;
- There is a lot of macho too, guys don’t like to admit they got hurt and are in pain, they also don’t want to be teased and never report”;
- “The company just fired people when they got hurt or sick. Most people just shut up. They know there are always new people who wants jobs”;
- “I work on the cut floor and have immense pain in my neck, shoulder and arm but my supervisor won’t move me. Some days I cry the whole time, I use muscle cream but the pain continues. I am still getting hospital bills from a previous work injury”.

As found internationally, no poultry industry specific occupational disease statistics are available in SA, but in general there is a very high incidence of noise-induced hearing loss and very low incidence of ergonomically related compensable diseases across all sectors [84].

NIOSH, in one study, reported that 57% of all poultry workers suffer from some ergonomic conditions and added that 42% suffer from CTS, with 81% of the tasks with hand activity above the ACGIH action limit [85]. In the U.S., poultry abattoir processing workers have consistently suffered illness at twice the national average and in 2004, more than 15% of all abattoir workers reported days off work or sought medical care. During 2004 the U.S. poultry industry had the sixth highest injury and illness rate for the year [86].

## 2. Methods

During 2014 we sourced PubMed, Medline and Science Direct for studies up to 2014, in any language relating to ergonomic and physical health impacts on poultry abattoir processing workers using the following terms: ergonomic impacts poultry abattoir processing, physical impacts poultry workers, WRULDs poultry abattoir processing workers, MSD poultry abattoir processing, occupational exposure ergonomic hazards, noise poultry abattoir processing, cold poultry abattoir processing. Studies relating to the impact on poultry abattoir workers health, symptoms and disease were included. Tables reflecting these impacts were created taking into consideration country, year, population and sample size, disease or symptoms, as well as contributing and associated causation factors. The search also included grey literature from institutes, corporations, international and governmental agencies using the following keywords: occupational health legislation, poultry abattoir processing worker health, occupational disease statistics, management occupational hazards and management physical hazards. Examples of the websites are: ILO [87], HSE [88], DoL [89], UN [90], SAPA [91] and NIOSH [92]. Although auxiliary activities at poultry processing plants, such as laboratories, engineering workshops, laboratories, water treatment plants, boiler plants and rendering plants and waste disposal, may also have ergonomic and physical hazards that impact on the workers, they were not included in this review. 

### Ethical Statement

It needs to be placed on record that this article forms part of a broader study and has been approved by the Tshwane University of Technology (TUT) Ethics Committee (reference number REC2012/08/005).

## 3. Results

Literature relating to physical and ergonomic impact on poultry processing were found dating back to the 1970s, which highlights the fact that studies related to physical and ergonomic risks in poultry abattoir processing have been conducted over the decades. We could find no relevant scientific studies specifically to physical and ergonomic impacts on SA poultry abattoir processing workers; however, available studies did focus on immunological and respiratory hazards. Most studies originated from the US and to a much lesser extent from Europe, Asia and South America.

### 3.1. Occupational Hazards from Physical Agents

Ideally, this review would provide a summary of the latest data on occupational diseases and conditions related exposure to physical agents and ergonomic stressors. Unfortunately, poultry abattoir associated information about these diseases are widespread and fragmented. Nevertheless, Table 2 provides a summary of the effects of physical agents.

### 3.2. Occupational Ergonomic Hazards

In 2011/2012, MSD represented 40% of all cases in the UK [93]. Approximately 1.2 million UK workers suffered from work related illness, with an overall annual total work-day loss of 28.2 million days and an estimated cost of injuries and ill health amounting to £14.4 billion per year across all sectors [65] In Northern Carolina, Department of Labor reports classify the differences between some poultry slaughter actions or tasks due to mechanical and manual operations and the potential to cause ergonomic stress leading to MSD in large and small scale poultry production, as reflected in Table 3 [60,94].

**Table 3:** Generic poultry processing phases indicating mechanical and manual actions that may contribute to ergonomic stress leading to musculoskeletal disorders.

Action or Task	Large Scale—Mechanical Process		Small Scale—Mostly Manual	
	Mechanical Line	Ergonomic Hazard	Manual or Hand Operated Line	Ergonomic Hazard
Off loading	Easy load system		By hand	Yes
Live shackling	Hang birds by hand	Yes	Hang birds by hand	Yes
Stunning	In line electrical water bath		Dry method held by hand	Yes
Bleeding	Bleeding follows mechanical neck cutting		Manual neck slitting bird placed in bleed cones	Yes
De-feathering	In line de-feathering machine & final manual de-feathering	Yes	Handheld or small scale de-feathering apparatus	Yes
Head, feet removal	In-line mechanical head pulling & hock cutting		Neck cut off with scissors or knife	Yes
Vent cutting & cloaca removal	Pneumatic vent drill, knife or scissors	Yes	Manually	Yes
Abdominal slitting	In line opening cutter		Knife or scissor	Yes
Evisceration	In line evisceration machine		Manual evisceration spoons	Yes
Crop & oesophagus removal	In line cropping machine		Manual crop removal (pre-evisceration)	Yes
Separation of carcass & organs	Per hand or manually	Yes	Hand separation	Yes
Carcass rehang	Per hand	Yes	Per hand	Yes
Red & dirty offal separation	Automatic separators		Hand separation	Yes
Giblet harvesting separating gizzard from gut	Automatic separation of intestines & gizzard Clean gizzard	Yes	Hand separation and manual cleaning	Yes
Neck pulling	In line neck puller		Knife or scissor cut	Yes
Final inspection; Debris removal from carcass	In line vacuum machine		Hand held vacuum machine/tube	Yes
Final washing	Automatic inside outside washer		Hand wash by spray	Yes
Chilling	Spin/ air chillers		Commercial type freezers—lifting	Yes
Portioning	In line cutting machine		Manual cutting	Yes
Packing	Automatic weighing & hand sorting	Yes	Packing and sorting by hand	Yes
Individual quick freeze	Gyro freezer		Freezer or blast freezer—lifting	Yes

Ergonomic conditions developed due to disorders of the muscles, nerves, tendons, joints, cartilage, supporting structures of the upper and lower limbs, neck and lower back which are caused, precipitated or exacerbated by sudden exertion or prolonged exposure to physical factors such as repetition, force, vibration, or awkward posture and disorders are classified in terms of these causes [33,95,96]. Table 4 indicates typical MSDs and whether the origin of the condition relates to the muscles, nerve or tendons as well as the effects that occur in poultry abattoir processing workers [17,96,97,98,99,100,101,102].

**Table 4:** Musculoskeletal disorders from repetitive and manual tasks.

Major Effects	Disorder Type	Description
Myalgia	Muscle	Muscle pain
Chronic myofascial pain syndrome	Muscle	Chronic muscle pain
Tendinitis	Tendon	Inflammation of a tendon for instance in elbow associated with repetitive tasks
Rotator cuff injuries	Tendon	Tendon inflammation in the shoulder
Epicondylitis (tennis elbow)	Tendon	Irritation of tendons attaching epicondyle due to forceful wrist movements
Tendosynovitis	Tendon	Inflammation of a tendon and its synovial sheath for instance in wrist, hands or fingers
Carpal tunnel syndrome	Nerve	Swelling or entrapment of the median nerve in the wrist
Hand arm vibration syndrome	Vessel	Blood vessel and nerve damage in hands and wrists; Compression of the median nerve of the forearm
Raynaud syndrome	Vessel	Insufficient blood supply characterised by blanching effect, loss of sensation and movement

Table 5 provides a non-exhaustive list of ergonomic and physical hazard related effects and disease relating to poultry abattoir workers, which includes information on country, study design and findings.

**Table 5:** A non-exhaustive summary of ergonomic effects, conditions and disease relating to poultry processing.

Research Study	Main Findings	Research Information	Reference
**Musculoskeletal disorders (MSD)**			
Taiwan: General cleaning workers	Cleaning workers at risk of: Musculoskeletal discomfort, pain reported by 90% cleaners in: Hand, wrist 42%; Shoulders 41% Low back 38%; Elbows 33%	Workers in awkward positions Associated contributing psychosocial factors: Time pressure/Speed of work Production targets	Chang, 2012 [103]
Canada: Female workers	MDS in women MSD may lead to accidents, efficiency decrease	Contributory factors: Poor tool design/Tool use/Force exertion	Messing, 1997 [104]
U.S.: 13 Female poultry processors	Mechanical deboning: Muscular activity significantly higher during: Repetition, Extreme wrist postures, Peak acceleration Moderately reduced some peak forces Manual cut: Extreme wrist postures—more frequent in cutting	Contributing factors: Force requirements Work postures Repetitive movements Increase muscle activity during cutting the most strenuous part of manual deboning: Different muscle groups used	Juul-Kristensen, 2002 [105]
U.S.: 200 Poultry abattoir processing workers <35 years old	MSD prevalence	Female workers show higher risks than male workers Worker age < 35 years No significant absenteeism & no medical care sought	Quandt, 2006 [106]
U.S.: 319 Female poultry abattoir processing workers	Three fifths reported musculoskeletal symptoms Greater job demands shows a greater MSD prevalence & depressive symptoms Lower skill variety & lower job control shows a greater MSD & depressive symptoms prevalence	Job demands included: Heavy load, awkward posture, greater psychological demands Greater support & with management (supervisor’s authority & safety climate) fewer depressive symptoms	Arcury, 2014 [107]
U.S.: Poultry abattoir processing workers	57% diagnosed with at least one MSD or symptom 39% reported hand symptoms Prevalence: Hand/ wrist tendonitis 8% Trigger finger 4% Ganglion cysts 3% Traumatic Injuries: Nerve damage in hands 72% showed abnormal results with the presence of median mono neuropathy in hands in 79% Damage degree: Mild 25%; Moderate 60%; Severe 15% 2009–2012 Incidence higher than the U.S. average	Killing 90 birds per minute; 160,000 per day Forceful repetitive work with knife use 50% participants were obese—BMI **^1^** > 30 58% workers indicated the use of cutting tools 47% worked overtime on weekly basis 41% of the non-overtime workers did job rotation—lower prevalence 43% visited plant medical clinic reporting symptoms of: Pain; Burning; Tingling; Symptoms of numbness in hands & wrists 41% of workers worked at levels above the ACGIH TLV for hand activity & force At baseline study 36% and at follow up 32% were performing tasks above the ACGIH TL	Musolin, 2014 [85]
Denmark: 3123 workers across 19 industries & poultry abattoirs	Prevalence of: Hand wrist pain; Tendinitis; Extensor tendinitis	Contributing factors: Repetitive work Force	Thomsen, 2007 [108]
U.S.: 291 poultry processing females	MSD symptom differences observed between poultry processing women & controls Upper extremity and neck symptoms 2.4 times higher	Contributing factors: Rapid line speed/Repetitive work Potential magnitude of upper extremity morbidity among women in poultry	Lipscomb, 2007 [109]
U.S.: 291 Female poultry workers	Early MSD onset Continued exposure cause rapid onset among women	Highly repetitive work Psychosocial variables included: Work organisation factors, Prevalence of other medical conditions, Depressive symptoms, Children at home, Hand intensive home activities, Age, Obesity, Job insecurity Complex relationships exists between physical work & psychosocial factors	Lipscomb, 2008 [110]
New Zealand: 237 workers, union, safety personnel management at 28 meat processing sites	Knife dullness cause increase use of force Greater risk of MSD of the neck & upper limbs	Table height, knife handle guarding & use of gloves play a role during cutting Highest incidence of MSD in meat processing & poultry processing accounting for over 50% of compensation costs for the sector	Tappin, 2008 [111]
Portugal: 50 meat packers	MSD diagnosed in 42% 88% of workers had two or more conditions Higher female prevalence 39% *vs.* 12% in males MSD disease/syndrome incidence: CTS (9) Osteoarthritis in fingers (5) Lateral epicondylitis (4) de Quervains disease (2) Guyon canal syndrome (2) Radial tendinitis (1) Tendoperiostitis of great palmar nerve (1) Tendosynovitis—distal in upper extremity joints (1)	Vibration from hand tools Repetitive work Precision movements Nine workers contributed to 446 days off work for the year	Sarranheira, 2008 [112]
Brazil: 290 poultry abattoir processing workers	67% suffered discomfort, pain in: Shoulders 63%; Neck 43%; Spine 36%; Forearms 31%; Arms 29%; Wrists 26%; Hands 26%	88% engaged in repetitive tasks 61% used hand tools 54% workers experienced cold	Tirloni, 2012 [113]
Brazil: 6000 poultry abattoir processing workers	MSD prevalence	Associated with: Repetitive tasks/Cold exposure/Production increase/pace	Buzanello, 2012 [56]
U.S.: 403 Poultry abattoir processing workers	More than 35% workers reported: Workers suffered from back, wrist & hand symptoms lasting more than 1 day Greater pain occurrence in overtime workers	Contributing factors: Rapid work pace, repetitive motions Poultry workers reported more wrist & elbow symptoms More symptom prevalent in overtime workers	Schulz, 2012 [114]
**Back and arm discomfort/pain**			
U.S.: 699 poultry workers	Back & arm discomfort and pain	Women were more susceptible	Stuart-Buttle, 1994 [115]
U.S.: 516 poultry workers	Low back pain in 17% (*n* = 89)	May negatively impact long-term	Rosenbaum, 2013 [116]
U.S.: 518 poultry abattoir processing workers	Back pain	Management commitment, awkward posture; repeated movements predicted. Low job control, high psychological demands elevated among poultry abattoir processing workers	Grzywacz, 2012 [117]
**Epicondylitis**			
U.S.: 518 poultry abattoir processing workers	Epicondylitis	Awkward posture; repeated movements predicted Low job control, high psychological demands elevated among poultry abattoir processing workers Workers exposed to work organisation hazards that contribute to occupational health disparities	Grzywacz, 2012 [117]
U.S.: 516 poultry abattoir processing workers	Epicondylitis in 6%	Increased prevalence after age 40 May negatively impact long-term exposure	Rosenbaum, 2013 [116]
U.S.: 234 Female poultry abattoir processing workers	Epicondylitis prevalence	Awkward posture and decision latitude were associated with epicondylitis Work organization factors may affect workers health	Arcury, 2014 [118]
**Rotator cuff syndrome**			
U.S.: 518 poultry abattoir processing workers	Management commitment, awkward posture; repeated movements predicted Rotator cuff syndrome	Low job control, high psychological demands elevated among poultry abattoir processing workers Workers exposed to work organisation hazards that contribute to occupational health disparities	Grzywacz, 2012 [117]
U.S.: 516 poultry abattoir processing workers	Rotator cuff syndrome 15% (*n* = 76)	Increased prevalence after age 40 May negatively impact long-term	Rosenbaum, 2013 [116]
U.S.: 234 Female poultry abattoir processing workers	Rotator cuff syndrome	Rotator cuff syndrome associated with awkward posture, psychological demand Work organisation factors affect health	Arcury, 2014 [118]
**Impingement syndrome**			
Denmark: Poultry workers employed 1986–1993	Impingement syndrome (IS) prevalent Physical examination revealed signs of subacromial impingement in the corresponding shoulder	Contributing factors contributing: Repetition; Force; Complicated movements; Shoulder intensive work; is diagnosed if symptoms were present for 3 months with subacromial impingement signs	Frost, 1999 [119]
**Carpal Tunnel Syndrome**			
Taiwan: 207 meat packers	CTS prevalence: Workers performing repetitive tasks 41% Workers exposed to cold & performing repetitive tasks 37%	Contributing factors: Force exertion Repetitive wrist movements Cold exposure	Chiang, 1990 [120]
U.S.: 30 male poultry abattoir processing workers	CTS from use of tools in deviated, angular wrist positions Pinch strength decrease	Strength degradation ranged from 14% to 43% Effect on maximum voluntary pinch strength: Least effect on: Natural deviation, radial deviation (smallest effect), Greatest effect on: Ulnar deviation, dorsiflexion & palmar flexion	Imrhan, 1991 [121]
U.S.: 157 poultry processors	50% workers had 3 or more of 22 conditions The average worker had 5 to 6 abnormal findings Major conditions/symptoms: Impaired pinch, decreased finger sensitivity, Hand/ finger numbness	Contributing factors: Vibration and repetitive tasks	Young, 1995 [122]
U.S.: 1591 Poultry abattoir processing workers	CTS prevalence: Deboning tasks dominant hand statistical significance: Reference group 2% Non-deboning abattoir workers 5% Deboning processing workers 8%	Associated with: Repetitive deboning tasks High-force and high-velocity manual work	Frost, 1998 [123]
India: Review CTS in food workers including poultry	CTS prevalence significant in: Abattoirs; Poultry processing; Meat processing; Frozen food workers; Packaging industry	Contributing factors: Prolonged repetitive hand intensive activities; Forceful exertions; Awkward or static postures; Vibration; Cold; Localised mechanical stress	Jagga, 2011 [124]
Taiwan: General cleaning workers (non-poultry)	Wrists at extreme angles of ulnar and radial deviation increased risk of CTS development	Associated psychosocial factors: Time pressure; Pace of work; Production targets	Chiang, 2012 [103]
U.S.: 287 poultry abattoir processing workers	CTS prevalence 8.7% higher in poultry processing Lower CTS trends in: Packing, sanitation & chilling workers	Repetitive & strenuous hand movement	Cartwright, 2012 [125]
U.S.: 318 Poultry abattoir processing workers	42% workers met the CTS criteria CTS prevalence 10% 47% females; 28% males Degree of CTS: Mild 20%; Moderate 60%; Severe at 21% 15% or workers reported absenteeism	50% participants were obese—BMI > 30 The mean age of CTS sufferers was 42 years 58% workers indicated the use of cutting tools 47% worked overtime on weekly basis 41% work at levels above ACGIH TLV for hand activity & force 41% did job rotation (non-overtime workers)	Musolin, 2014 [85]
U.S.: Latino poultry abattoir slaughtering & processing workers (106 wrists)	Based on 106 wrists, the 1-year incidence of CTS was higher in poultry processing workers (20%) than non-poultry manual workers (12%)	Contributing factors: Wrist position; Repetitive & strenuous nature of poultry processing work Poultry workers has significantly higher chance for CTS development	Cartwright, 2014 [126]
U.S.: 234 Female poultry abattoir processing workers	Carpal tunnel syndrome prevalence	Awkward posture & psychological demand & decreased skill variety & job control were related to CTS Work organisation factors important for musculoskeletal & neurological injury	Arcury, 2014 [118]
**Raynaud Syndrome: Finger sensitivity**			
France: 17 poultry abattoirs: 1474 workers	Raynaud Syndrome Finger sensitivity	More common in women Contributing factors: Cold environment Repetitive tasks; Arm exertion; Vibrating tools; Plastic gloves Aggravated by Infrequent breaks in cold areas	Kaminski, 1997 [52]
**Callosities, calluses, knuckle pads**			
U.S.: 41 Live bird hangers	Knuckle pads were observed in 56% (23) chicken hangers	Repeated striking, knocking & sliding of knuckles against metal	Richards, 1987 [127]
**Poverty**			
U.S. 2009: Poultry abattoir processing workers	Female poultry workers displayed a 36% PHRQoL **^2^**& moderate to high incidence of MSD	Link between MSD & PHRQoL	Armstrong, 1982 [128]
**Job stress** & **Strain**			
U.S.: Poultry inspectors Comparison of 4 groups: Full- and Part-time inspectors; Rotating relief inspectors; Supervisory group	Full-time inspectors had the highest frequency rates for 17 health symptoms Followed by Rotating relief inspectors with 9 most prevalent health complaints: Respiratory; Skin; Musculoskeletal; Gastrointestinal; Visual complaints, Job stress & strain	Full-time inspectors: Highest job stress & poorest work environment scores Supervisor social support lowest for full-time inspectors Rotating relief inspectors had least support from others at work Psychological & behavioural strain highest for full-time inspectors	Wilkes, 1981 [129]

**^1^** BMI—Body mass index; **^2^** Low physical related quality of life.

## 4. Discussion

Literature reflected in Table 3 and Table 5 reveals that physical as well as ergonomic hazards can cause several symptoms, effects and diseases in poultry abattoir processing workers.

### 4.1. Noise

Noise levels in poultry production may reach levels well in excess of the OEL for noise; for instance levels during primary processing (87 dB(A)), meat cutting and processing (90 dB(A)), packaging including hoppers (95 dB(A)), blast chillers (107 dB(A)) are major sources of exposure. Noise level may vary depending on level of production, condition of equipment, processes involved and type of noise caused and lead to occupational related noise-induced hearing loss, reproductive impact, lowered birth rate and increase in blood pressure, amongst others [45,46,47,48,49].

### 4.2. Vibration

Hand-Arm Vibration (HAV) is defined as the transfer of vibration from a tool to a worker’s hand and arm. The amount of HAV is characterised by the acceleration level of the tool when grasped by the worker and in use [97,130]. Vibrating equipment, for instance Whizard**^®^** knifes, causes an interaction between vibration, repetitive tasks, force and cold causing Hand-Arm vibration syndrome (HAVS), which is aggravated in the presence of cold and by performing repetitive tasks [52,129]. 

### 4.3. Cold

To ensure a quality product, production temperatures are set for certain phases of production such as quick freeze areas, package, cold stores and dispatch areas [55,131]. The optimum temperature range for humans varies between 13 and 24 °C, with production temperatures recorded well outside this range [17,55,131,132]. Cold exposure also impact on and aggravate vibration and ergonomic effects [56,113].

### 4.4. Ergonomic Hazards

Despite advances, automation and improved work procedures, the poultry industry is labour and hand intensive with many tasks being repetitive in nature, requiring force and involving processes such as receiving and live hanging of birds, which may be up to 35 birds per minute, slaughtering, lifting, shoving, twisting, reaching, hanging, carrying, processing, value adding, packaging and shipment all being repeated several times a day [20,133]. Ergonomic hazards affect hands, wrists, arms, shoulders, the neck and back, with workers repeating thousands of repetitive actions, twisting and forceful motions sometimes completing more than 2000 cuts per shift or hanging more than a thousand birds or carcases during a shift. Workers complete short job cycles of under 10 s repeating the same apparent trivial movements sometimes up to 30,000 times a day—repeating the same task for eight to ten hours per shift during a typical workday with limited breaks, sometimes performing the task in awkward or static postures [60,134,135,136]. Poor facilities, machine and tool design, faster production lines and greater production output places increasing physical stress and demand on workers [20]. Disorders are classified mainly as WRULD, which includes CTS, tendinitis, rotator cuff injuries, epicondylitis, trigger finger, muscle strain, occupational over exertion or overuse syndrome (OOS), CTS and neck and back injuries. All conditions are associated with discomfort and pain and develop over weeks, months and/or years leading to worker absence to recover [52,54,62,68,134,135,137]. In epidemiological studies, disease must consistently be associated with an occupational health hazard, but in MSD, it depends on the individual’s interaction with the dimensions of the work site and task, causing scepticism of existence of illness due to repetitive movements leading to increased worker suffering [138]. Mechanisation and automation to achieve higher production in the poultry industry could not replace knife use, a very essential part in cutting, removal of fat or skin, trimming and processing. Considerable attention has been given to knife design but using knifes implies the use of force and forceful exertions which assist in MSD development [139,140].

### 4.5. Production Line Optimisation

Workers have no control over the line speed and cannot stop to rest or take breaks, a fundamental principal in ergonomics [141]. The editor of weblog GIGjob profiles, reported an interview conducted by an anonymous 30 year old worker who stated that “Taking regular work breaks is not always so easy. If we are not done with the truckload of chickens, we cannot leave work at the end of our shift, we are slave...; you just have to be very fast. You’re not always working safely because you have to keep up with the production line. The managers always want more production in less time” [142].

In SA, some high throughput abattoirs slaughter 350,000 to 400,000 birds per 10 h shift [143]. In the U.S., production line speeds of 70 birds per minute was increased to 120 birds per minute and the increase in line speed lead to greater productivity and profit, but not to safer and healthier poultry processing plants. In view of this, the industry still has one of the highest rates of occupational injuries and illness, at rates of more than twice all manufacturing sector averages [20,144]. On production line speeds, U.S. poultry workers stated [20]:

- “I came to Arkansas in 1995 and at the time we did 32 birds a minute. I came back and it was 42. People can’t take it”.
- “The lines are too fast. The work speed is for machines and not humans. You have to work the knife too hard. That is when pain starts”.

After complaints from the Southern Poverty Law Centre, OSHA found workers suffered MSD at a U.S. poultry producer and that the employer failed to record and properly manage the injuries and medical treatment of injured employees, failed to refer workers to physicians and discouraged them from seeking medical attention. The employer received 11 citations carrying $102,600 in total fines including two more serious general-duty-clause citations for alleged MSD hazards, carrying penalties of $14,000 for failing to provide a safe and healthy work environment [145]. Professor Tom Armstrong, who studied the prevalence of MSD in poultry abattoirs, states “It is highly unlikely that any poultry plant could go consecutive years without incidence of the MSD conditions, carpal tunnel syndrome and tendonitis” [73].

## 5. Conclusions

Factors such as individual susceptibility, duration, frequency and intensity of exposure to ergonomic and physical hazards play an important role in impacting on worker health and well-being, significantly causing conditions that lead to occupational disease, discomfort and pain, with females at greater risk than males. It also impacts on workers through impoverishment, affecting society at large [25,62,133,146,147]. Occupational disease can impose enormous costs and increase health costs and can impact on producers, reduce productivity and work capacity [72,80,148,149]. Globally, work related disease, including accidents, resulted in an annual 4% loss in global GDP, or about U.S. $2.8 trillion, in direct and indirect costs. In the U.S. alone, $32 billion was paid by prosecuted enterprises across all sectors [86]. Workplace illness cost the UK £8.4 billion and in the EU, the cost of work-related diseases has been estimated to be at least €145 billion per year [150]. 

In SA, the combined compensation for occupational diseases and occupational injuries indicates an escalating compensation pattern from 886,511 for 2006/2007, to 934,834 in 2010/2011 [151]. Internal and external reporting mechanisms often fail workers who are ill informed and not properly trained.

Employers are legally compelled to provide a healthy work environment by assessing the health risk workers are exposed to and to implement and manage control systems to prevent occupational disease. They achieve this by using the services of occupational hygienists to assist with anticipating, recognition, evaluation and control of occupational hazards. Employers should implement best practices in the design of controls and in prevention programmes [152]. Controls may include the redesign of tasks, processes and tools, administrative controls through job rotation, reduction in shift duration, broadening of work content thus adapting the work environment and not the worker.

Where possible, it is best to alleviate the risk by workstation design, *i.e.*, adjustable height, the layout of conveyors and equipment, design of and equipment to avoid the need for workers to adopt awkward postures. Most work, especially forceful cuts, or gripping and lifting tasks should be done within the “comfortable reach zone” and with the wrist and elbow close to the neutral position, repetitive handling should be done in a zone 450 mm in front of the body. Knives should have secure grips and be sharp as this reduces force; unfortunately most gloves affect the grip negatively leading to workers applying more force. Packaging should be designed to limit lifting or picking up, care should be taken to avoid cold draughts on the shoulders and necks of workers and machines should be maintained to reduce noise levels [55,60,133,153]. To reduce risks further job rotation could be a positive strategy, as by moving workers between different tasks which require different grips and different muscle groups, prolonged repetition is avoided. Rest breaks are important where highly paced, repetitive work is done and productivity falls quite quickly after the start of the shift and scheduled breaks should be timed so that workers get a rest before their arms or shoulders become fatigued. Workers must receive training on handling tools, the importance of breaks, wearing of personal protective equipment to protect against cold and noise. It is also important for workers to understand the need to take scheduled breaks and to use them as an opportunity to rest and recover. If exercise is introduced, it is important the exercises are designed by someone with sound knowledge of bio-mechanics such as a physiotherapist or ergonomist. ULD risks are higher where workers have little or no control over the pace at which they work [19,20,55,94,133,154].

To ensure these measures are effective, occupational health practitioners and occupational medicine practitioners are used to prevent, diagnose and treat occupational disease by instituting medical surveillance of workers [133,135]. Employers must promote early reporting of symptoms and set up mechanisms to collect relevant data and for the review of records, complaints, absenteeism, clinic visits to establish links between data obtained and specific tasks [155,156]. Baseline medicals must be conducted to establish a base against which changes can be evaluated through routine medical examination. Employees with any work related conditions must be promptly evaluated and appropriate treatment and follow-up provided. Work exposure trend analysis and periodic symptom surveys can be conducted among workers [80,94,148,157]. Workers must be informed about the occupational hazards, instructed on measures to protect their health and trained on all related preventative aspects [148,157,158,159]. Workers, as well as management, should play an active part in this by participating and cooperating in the ergonomics programme, undergo training and applying the principles in their everyday work and promptly report any condition to the company clinic [97,135,155,156,160,161].

On a managerial level, effective control and management can only be achieved through strong and visible employer leadership, commitment and support, worker involvement and effective training [85]. Employers should develop a process to systematically address ergonomic and physical related occupational health hazards and incorporate them into their existing health (and safety) programmes by [85,161]:

- continual communication on the importance of worker health at all levels;
- assigning and communicating the roles and responsibilities for the different aspects of the ergonomic and physical process to managers, supervisors and employees;
- committing adequate resources to the ergonomics and physical process;
- integrating health (and safety) concerns into production processes and production improvements.

Limitations encountered were the lack of specific research at SA poultry abattoirs to present some indication of conditions and the confusion regarding the use of terminologies, or the use of outdated terms, is noteworthy. Compensation criteria varies from country to country [40,62,63,95] and compensation statistics in general do not include specific incidence for poultry processing workers [63,84].

There is a need to perform more research about physical and ergonomic hazards and their impact, especially in SA and to develop management tools specific to the poultry industry, as well as a need for the industry to grasp the extent of exposure and to implement cost effective controls to improve worker health and well-being. In addition, bridging organisations such as industry organisations can provide a platform for building trust, making sense, provide information, instruction and training, vertical and horizontal collaboration and conflict resolution. Meaningful knowledge is likely to result in concept development, attitudinal change and positive behaviour.
